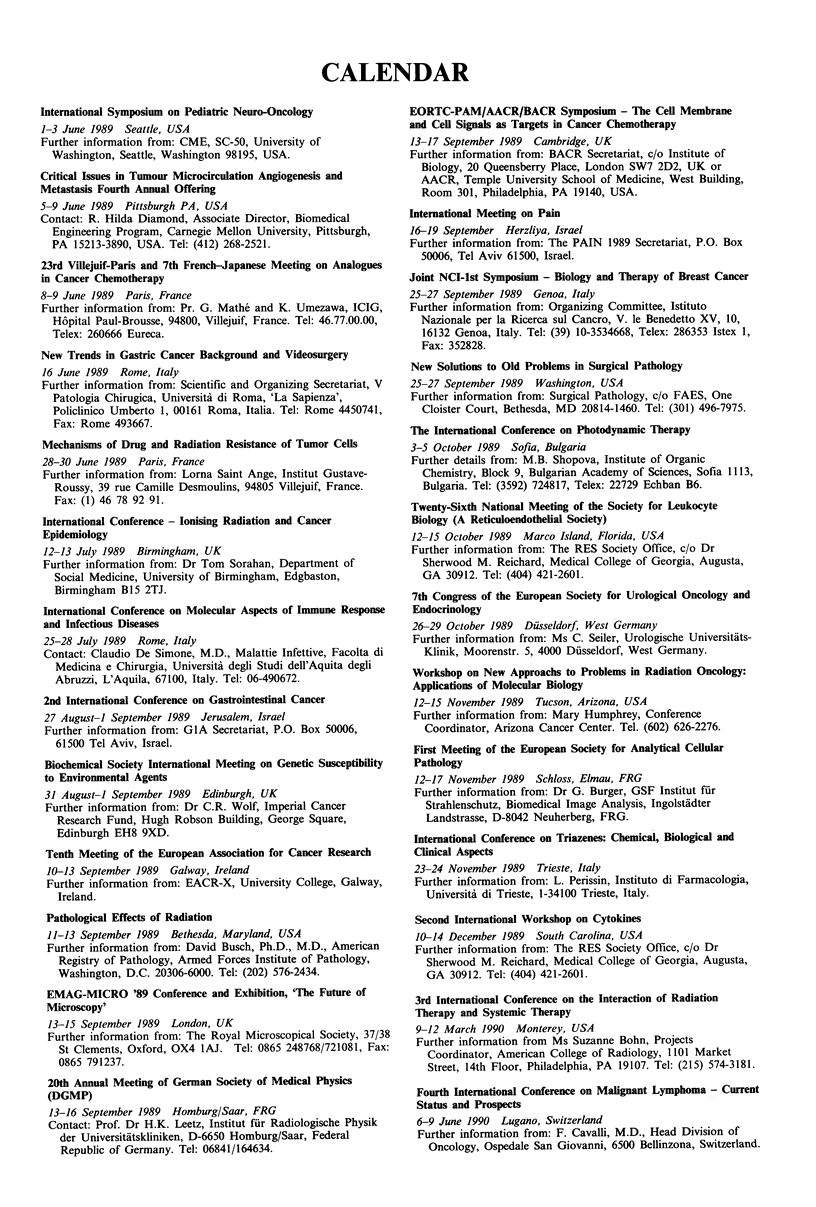# Calendar

**Published:** 1989-05

**Authors:** 


### CALENDAR

**International Symposium on Pediatric Neuro-Oncology**

*1–3 June 1989 Seattle, USA*

Further information from: CME, SC-50, University of Washington, Seattle, Washington 98195, USA.

**Critical Issues in Tumour Microcirculation Angiogenesis and Metastasis Fourth Annual Offering**

*5–9 June 1989 Pittsburgh PA, USA*

Contact: R. Hilda Diamond, Associate Director, Biomedical Engineering Program, Carnegie Mellon University, Pittsburgh, PA 15213-3890, USA. Tel: (412) 268-2521.

**23rd Villejuif-Paris and 7th French–Japanese Meeting on Analogues in Cancer Chemotherapy**

*8–9 June 1989 Paris, France*

Further information from: Pr. G. Mathé and K. Umezawa, ICIG, Hôpital Paul-Brousse, 94800, Villejuif, France. Tel: 46.77.00.00, Telex: 260666 Eureca.

**New Trends in Gastric Cancer Background and Videosurgery**

*16 June 1989 Rome, Italy*

Further information from: Scientific and Organizing Secretariat, V Patologia Chirugica, Università di Roma, 'La Sapienza', Policlinico Umberto 1, 00161 Roma, Italia. Tel: Rome 4450741, Fax: Rome 493667.

**Mechanisms of Drug and Radiation Resistance of Tumor Cells**

*28–30 June 1989 Paris, France*

Further information from: Lorna Saint Ange, Institut Gustave-Roussy, 39 rue Camille Desmoulins, 94805 Villejuif, France. Fax: (1) 46 78 92 91.

**International Conference – Ionising Radiation and Cancer Epidemiology**

*12–13 July 1989 Birmingham, UK*

Further information from: Dr Tom Sorahan, Department of Social Medicine, University of Birmingham, Edgbaston, Birmingham B15 2TJ.

**International Conference on Molecular Aspects of Immune Response and Infectious Diseases**

*25–28 July 1989 Rome, Italy*

Contact: Claudio De Simone, M.D., Malattie Infettive, Facolta di Medicina e Chirurgia, Università degli Studi dell'Aquita degli Abruzzi, L'Aquila, 67100, Italy. Tel: 06-490672.

**2nd International Conference on Gastrointestinal Cancer**

*27 August–1 September 1989 Jerusalem, Israel*

Further information from: G1A Secretariat, P.O. Box 50006, 61500 Tel Aviv, Israel.

**Biochemical Society International Meeting on Genetic Susceptibility to Environmental Agents**

*31 August–1 September 1989 Edinburgh, UK*

Further information from: Dr C.R. Wolf, Imperial Cancer Research Fund, Hugh Robson Building, George Square, Edinburgh EH8 9XD.

**Tenth Meeting of the European Association for Cancer Research**

*10–13 September 1989 Galway, Ireland*

Further information from: EACR-X, University College, Galway, Ireland.

**Pathological Effects of Radiation**

*11–13 September 1989 Bethesda, Maryland, USA*

Further information from: David Busch, Ph.D., M.D., American Registry of Pathology, Armed Forces Institute of Pathology, Washington, D.C. 20306-6000. Tel: (202) 576-2434.

**EMAG-MICRO '89 Conference and Exhibition, 'The Future of Microscopy'**

*13–15 September 1989 London, UK*

Further information from: The Royal Microscopical Society, 37/38 St Clements, Oxford, OX4 1AJ. Tel: 0865 248768/721081, Fax: 0865 791237.

**20th Annual Meeting of German Society of Medical Physics (DGMP)**

*13–16 September 1989 Homburg/Saar, FRG*

Contact: Prof. Dr H.K. Leetz, Institut für Radiologische Physik der Universitätskliniken, D-6650 Homburg/Saar, Federal Republic of Germany. Tel: 06841/164634.

**EORTC-PAM/AACR/BACR Symposium – The Cell Membrane and Cell Signals as Targets in Cancer Chemotherapy**

*13–17 September 1989 Cambridge, UK*

Further information from: BACR Secretariat, c/o Institute of Biology, 20 Queensberry Place, London SW7 2D2, UK or AACR, Temple University School of Medicine, West Building, Room 301, Philadelphia, PA 19140, USA.

**International Meeting on Pain**

*16–19 September Herzliya, Israel*

Further information from: The PAIN 1989 Secretariat, P.O. Box 50006, Tel Aviv 61500, Israel.

**Joint NCI-1st Symposium – Biology and Therapy of Breast Cancer**

*25–27 September 1989 Genoa, Italy*

Further information from: Organizing Committee, Istituto Nazionale per la Ricerca sul Cancro, V. le Benedetto XV, 10, 16132 Genoa, Italy. Tel: (39) 10-3534668, Telex: 286353 Istex 1, Fax: 352828.

**New Solutions to Old Problems in Surgical Pathology**

*25–27 September 1989 Washington, USA*

Further information from: Surgical Pathology, c/o FAES, One Cloister Court, Bethesda, MD 20814-1460. Tel: (301) 496-7975.

**The International Conference on Photodynamic Therapy**

*3–5 October 1989 Sofia, Bulgaria*

Further details from: M.B. Shopova, Institute of Organic Chemistry, Block 9, Bulgarian Academy of Sciences, Sofia 1113, Bulgaria. Tel: (3592) 724817, Telex: 22729 Echban B6.

**Twenty-Sixth National Meeting of the Society for Leukocyte Biology (A Reticuloendothelial Society)**

*12–15 October 1989 Marco Island, Florida, USA*

Further information from: The RES Society Office, c/o Dr Sherwood M. Reichard, Medical College of Georgia, Augusta, GA 30912. Tel: (404) 421-2601.

**7th Congress of the European Society for Urological Oncology and Endocrinology**

*26–29 October 1989 Düsseldorf, West Germany*

Further information from: Ms C. Seiler, Urologische Universitäts-Klinik, Moorenstr. 5, 4000 Düsseldorf, West Germany.

**Workshop on New Approachs to Problems in Radiation Oncology: Applications of Molecular Biology**

*12–15 November 1989 Tucson, Arizona, USA*

Further information from: Mary Humphrey, Conference Coordinator, Arizona Cancer Center. Tel. (602) 626-2276.

**First Meeting of the European Society for Analytical Cellular Pathology**

*12–17 November 1989 Schloss, Elmau, FRG*

Further information from: Dr G. Burger, GSF Institut für Strahlenschutz, Biomedical Image Analysis, Ingolstädter Landstrasse, D-8042 Neuherberg, FRG.

**International Conference on Triazenes: Chemical, Biological and Clinical Aspects**

*23–24 November 1989 Trieste, Italy*

Further information from: L. Perissin, Instituto di Farmacologia, Università di Trieste, 1-34100 Trieste, Italy.

**Second International Workshop on Cytokines**

*10–14 December 1989 South Carolina, USA*

Further information from: The RES Society Office, c/o Dr Sherwood M. Reichard, Medical College of Georgia, Augusta, GA 30912. Tel: (404) 421-2601.

**3rd International Conference on the Interaction of Radiation Therapy and Systemic Therapy**

*9–12 March 1990 Monterey, USA*

Further information from Ms Suzanne Bohn, Projects Coordinator, American College of Radiology, 1101 Market Street, 14th Floor, Philadelphia, PA 19107. Tel: (215) 574-3181.

**Fourth International Conference on Malignant Lymphoma – Current Status and Prospects**

*6–9 June 1990 Lugano, Switzerland*

Further information from: F. Cavalli, M.D., Head Division of Oncology, Ospedale San Giovanni, 6500 Bellinzona, Switzerland.